# Microstructural Evolution and Mechanical Properties of an Advanced γ-TiAl Based Alloy Processed by Spark Plasma Sintering

**DOI:** 10.3390/ma12091523

**Published:** 2019-05-09

**Authors:** David Wimler, Janny Lindemann, Helmut Clemens, Svea Mayer

**Affiliations:** 1 Department of Materials Science, Montanuniversität Leoben, 8700 Leoben, Austria; helmut.clemens@unileoben.ac.at (H.C.); svea.mayer@unileoben.ac.at (S.M.); 2 GfE Fremat GmbH, 09618 Brand-Erbisdorf, Germany; janny.lindemann@gfe.com

**Keywords:** spark plasma sintering, γ TiAl based alloys, TNM alloy, heat treatment, mechanical properties

## Abstract

Intermetallic γ-TiAl based alloys are innovative lightweight structural high-temperature materials used in aerospace and automotive applications due to already established industrial-scale processing routes, like casting and hot-working, i.e., forging. A promising alternative method of production, regarding manufacturing of near net-shape components, goes over the powder metallurgy route, more precisely by densification of TiAl powder via spark plasma sintering. In this study, gas atomized powder from the 4th generation TNM alloy, Ti-43.5Al-4Nb-1Mo-0.1B (in at.%), was densified and the microstructure was investigated by means of electron microscopy and X-ray diffraction. The sintered microstructure exhibits lamellar α_2_-Ti_3_Al /γ-TiAl colonies surrounded by globular γ- and ordered β_o_-TiAl phase. The coarse lamellar spacing stems from the low cooling rate after densification at sintering temperature. Against this background, subsequent heat treatments were designed to decrease the lamellar widths by a factor of ten. Accompanying, tensile tests and creep experiments at different temperatures revealed that the modified almost fully lamellar microstructure is enhanced in strength and creep resistance, where a small volume fraction of globular γ-phase provides ductility at ambient temperatures.

## 1. Introduction

The development of lightweight, high-temperature materials is still subject of worldwide research, because of the existing engine manufacturers’ demands to design and manufacture more efficient and eco-friendly propulsion systems [1,2,3,4]. In addition to new and improved design concepts, previous works have been shown that the use of intermetallic titanium aluminides based on the ordered γ-TiAl phase plays a decisive role in replacing heavy Ni-based alloys as turbine blades or turbocharger turbine wheels. Particularly in the temperature range of 600 to 800 °C, intermetallic γ-TiAl based alloys exhibit high specific tensile strength levels. As an example, the β-solidifying γ-TiAl based TNM alloy, a process-adapted 4th generation alloy with a nominal composition of Ti-43.5Al-4Nb-1Mo-0.1B (in at.%), can be regarded as an appropriate benchmark. The addition of Nb improves the oxidation resistance and along with Mo the creep properties [1,2]. In contrast to other Ti-Al alloy systems, the TNM alloy family is distinguished by its excellent hot-workability, i.e., it can be forged under isothermal as well as near conventional conditions, demonstrating its universal applicability [5]. Within the last decades, mainly melt-metallurgical processes have been developed for the production of high-quality starting material, which has subsequently been processed into structural components by casting or hot-forming associated with advanced heat treatments [5]. However, the TNM alloy was already selected as an experimental alloy to prove the feasibility for powder metallurgical (PM) processing routes, like additive manufacturing (AM) or spark plasma sintering (SPS), which enable the production of complex near-net shape components. Although these techniques have not reached industrial readiness up to now, encouraging results have been achieved [6,7,8]. Therefore, in the framework of this study, SPS was chosen to densify TNM powder by mechanical pressure and high intensity pulsed direct electric current within a graphite die. The shape of the graphite die even allows the sintering of a turbine blade, as already demonstrated by Couret et al. [6]. Another advantage of SPS is the possibility of sintering at different temperatures and times, whereby various microstructures can be adjusted [8]. However, one disadvantage in this respect is the utilization of natural convection for cooling, i.e., the cooling rate is limited and further depends on the sample geometry [6].

In general, a PM approach generates a chemical homogeneous starting condition for further applications. While alloying elements determine the mechanical properties of TiAl alloys, a further decisive factor in this context is the arrangement of the constituting phases in the microstructure. In the case of the multi-phase TNM alloy, the adjustment of the strength and ductility is depended on the volume fraction and morphology of the ordered hexagonal α_2_-Ti_3_Al phase (D0_19_ structure), the ordered body-centered cubic (bcc) β_o_-TiAl phase (B2 structure), as well as the ordered face-centered tetragonal γ-TiAl phase (L1_0_ structure) [9]. The γ-phase, which appears both globular and lamellar within the heat-treated TNM microstructure, increases the ductility at room temperature (RT), because the predominant deformation mechanisms are the glide of ordinary dislocation and mechanical twinning within the L1_0_ structure [10,11]. In contrast, the brittle β_o_-phase decreases the RT ductility, due to the lack of independent slip systems [11]. However, both α_2_/γ-colony boundary phases, i.e., the globular γ- and the β_o_-phase, suppress grain growth during thermal treatment, but affect the creep behavior negatively [10,12]. The creep and tensile strength can be further controlled by α_2_/γ lamellar spacing. These lamellar colonies form during moderate cooling from the disordered hexagonal α-phase, i.e., α→α_2_/γ, possessing the so-called Blackburn orientation relationship: (111)_γ_||(0001${)}_{\alpha_{2}}$, 〈110]_γ_||〈$11\bar{2}0\rangle {}_{\alpha_{2}}$. Due to the fact that this transformation is diffusion-controlled the lamellar spacing depends on the cooling rate, meaning that high cooling rates generate fine lamellar widths, which improve creep resistance and strength [10,13,14]. At elevated cooling rates, however, supersaturated α_2_-phase can be obtained at RT, tendering the formation of fine γ-lamellae, i.e., α_2_→α_2_/γ, in a subsequent aging step [15]. As a result, nano-lamellar structures can be achieved [15,16]. 

Therefore, two-step heat treatments have been developed for TNM alloys, obtaining a thermally stable microstructure during long-term exposure at service temperature, which provides balanced mechanical properties. The first heat treatment step takes place within the (α + β + γ) phase field region followed by air-cooling (AC) [9,15]. The microstructure consists of small volume fraction of globular β_o_- and γ-grains, as well as supersaturated α_2_-grains with a grain size well below 100 µm as described in ref. [17]. If the Al content of the TNM alloy is increased, an additional (α + γ)- and single α-phase field region arise [15], as shown in the quasi-binary phase diagram of the TNM alloy system reported in References [9,15,18]. Depending on the temperature and dwell time of the first heat treatment step and of the chemical composition, the adjustment of further types of lamellar microstructures, namely a nearly lamellar (NL) microstructure with traces of globular γ- (NLγ) and/or β_o_-phase (NLβ) or a fully lamellar (FL) microstructure, offering the highest creep resistance, as well as yield strength, can be accomplished [11,12]. The corresponding cooling and the subsequent second heat treatment step, which takes place in a temperature range above the service temperature and below the eutectoid temperature, followed by furnace cooling (FC), defines the average lamellar spacing. This so-called aging treatment must be selected in such a way that the optimum balance of mechanical properties is generated, in particular to obtain creep resistance at elevated temperature and to provide ductility below the brittle-to-ductile transition temperature [9]. 

In the case of SPS, current devices are able to consolidate, sinter and conduct a heat treatment step at the same time. However, unfortunately, the resulting coarse lamellar spacing, which is linked to the low feasible cooling rate in the course of natural convection, makes it impossible to optimally adjust the mechanical properties of the TNM alloy as described above. For this reason, the present study deals with the development of subsequent heat treatments on SPS manufactured samples in order to optimize their mechanical properties, extending the scope of the SPS PM route towards improved high-temperature applications. 

## 2. Experimental Procedure

The TNM powder used in the present study was produced via electrode induction melting gas atomization (EIGA) [19] by Nanoval GmbH & Co. KG, Germany, applying a Laval nozzle, using argon inert gas [20]. The ingots needed for this process were produced according to refs. [21,22] by GfE Metalle und Materialen GmbH, Germany. A powder fraction smaller than 150 µm was utilized for the SPS experiment, which was carried out on a SPS device of the type HP D 25 by FCT Systems GmbH, Germany, at the Technische Universität Bergakademie Freiberg, Germany. This SPS device has a cylindrical graphite die with an inner diameter of 80 mm, which corresponds to the sample cross-section at a height of 14 mm. The densification of the powder is reached under vacuum condition by mechanical pressure and a pulsed electric current. For details regarding the SPS process, the reader is referred to References [6,23].

The chemical composition of the TNM alloy after SPS is shown in Table 1. The concentrations of Ti, Al, Nb, and Mo were determined by means of X-ray fluorescence spectroscopy, while B was evaluated by inductively coupled plasma atomic emission spectroscopy. For analyzing the O content carrier gas hot extraction was used. The chemical analysis of the powder has shown the same results, with exception of the O content, which was 300 mass-ppm lower.

The TNM powder was sintered at 1300 °C for 3 min under an applied load of 50 MPa.

This dwell temperature was reached by using a heating rate of 50 K/min between RT and 1000 °C and, beyond this, the heating rate was decreased to 40 K/min in order to prevent a temperature overshooting. After a dwell time of 3 min, the sintered disc of the diameter 80 mm was cooled to RT with around 90 K/min via natural convection cooling of the SPS device.

Thermal treatments were conducted under atmospheric conditions using a high-temperature furnace of the type RHF 1600 from Carbolite, Germany. Subsequently, AC was performed. The heat treatments were carried out on cylindrical samples with a diameter of 11 mm and a length of 70 mm, which were eroded by electrical discharge machining from the sintered discs. Also the samples in as-SPS or heat-treated condition for microstructural examination by scanning electron microscopy (SEM) were taken from the center of the disc. The specimens were metallographically ground and electrolytically etched in accordance to Reference [24]. All SEM investigations were conducted on an EVO 50 by Zeiss, Germany, in back-scattered electron (BSE) mode at an acceleration voltage of 20 kV. Furthermore, the electrolytically etched samples, where the etching process reduced surface-near residual stresses, were investigated by X-ray diffraction (XRD) measurements using an AXS D8 Advanced diffractometer from Bruker, Germany. The Rietveld refinement [25] was used to quantify the fractions of the phases present, applying the Bruker software TOPAS 4.2, Germany.

Complementary, the SEM micrographs were analyzed via the image analysis software Stream Motion 1.9.3 from Olympus, Japan, to determine phase fractions and grain sizes. Thereby, the grain size corresponds to the equivalent circle diameter. For more details, the reader is referred to References [9,24].

The lamellar spacing of the α_2_/γ-colonies was examined by means of transmission electron microscopy (TEM) employing a Philips CM12 microscope, Germany, operating at an acceleration voltage of 120 kV. For these investigations, specimens of 3 mm in diameter were electrolytically thinned to electron transparency using a Tenu Pol-5 using the electrolyte A3 by Struers, Germany. The TEM images were taken along the 〈110〉 zone axis of the γ-phase in “edge-on” condition of the α_2_/γ-colonies. These TEM images were used to measure the width of α_2_ and γ lamellae to determine the average lamellar spacing.

Hardness was measured according to Vickers HV10 using a universal testing machine M4C 025 G3M from EMCO, Austria. Each hardness value was calculated as arithmetic mean value from at least five indents.

Quasi-static tensile tests at RT, 300 °C, 700 °C, and 800 °C were carried out on universal testing machines of the type Inspect 50-1 from Hegewald and Peschke, Germany, and AG-100 from Shimadzu, Japan, at the GfE Fremat GmbH, Germany. The tensile test specimens had a diameter of 5 mm and a gauge length of 25 mm. The initial strain rate of the tensile tests was always 10^−4^ s^−1^. At each temperature, two specimens of the as-SPS condition and the heat-treated variants were tested.

Creep tests of the heat-treated samples were performed at a temperature of 750 °C, using an initial constant load of 150 MPa employing creep testing machines TC30 and TC50 from AET Technologies, France. The creep samples had an initial diameter of 6 mm and an initial gauge length of 30 mm. Extensometer bars were used to determine the creep strain. Stable thermal conditions along the specimen length were guaranteed through monitoring and controlling the temperature by three attached thermocouples.

## 3. Results

### 3.1. Microstructure

**Figure 1:**
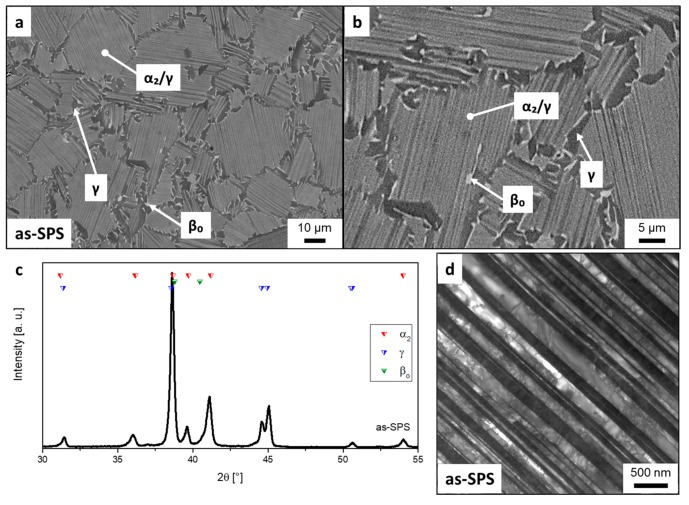
(**a**,**b**) NLγ+β_o_ microstructure of the as-SPS TNM alloy consisting of equiaxed lamellar α_2_/γ-colonies surrounded by globular γ- and β_o_-phase. Owing to the low cooling rate from the dwell temperature (1300 °C), the lamellar spacing is rather large. The SEM images were taken in BSE mode, thus globular γ shows a dark contrast, whereas the β_o_-phase appears in bright contrast. The α_2_-phase shows a contrast between those of γ and β_o_. (**c**) XRD spectrum of as-SPS sample. The phase fraction evaluated by applying Rietveld analysis is listed in Table 2. (**d**) TEM image of a lamellar α_2_/γ-colony in an as-SPS specimen in “edge-on” condition.

The TNM powder was spark plasma sintered at a dwell temperature of 1300 °C for 3 min under an applied pressure of 50 MPa, obtaining a dense specimen. The microstructure taken from the center of the as-SPS disc is shown in Figure 1a,b, exhibiting a NLγ+β_o_ microstructure consisting of equiaxed lamellar α_2_/γ-colonies surrounded by globular γ- (dark contrast) and β_o_-phase (bright contrast). The SEM images of the as-SPS condition were evaluated via image analysis. As a result, the globular γ-phase was estimated to be around 4 vol.% and for the β_o_-phase the value was below 1 vol.%. Additionally, XRD measurements (see Figure 1c) provide quantitative analysis regarding the crystallographic phase fraction, as listed in Table 2. The γ-phase fraction of the as-SPS sample is around 60 vol.%, which stems from both the γ-lamellar and the globular γ-phase. The lamellar spacing is rather large and the individual lamellae are already visible in the SEM image. This coarse spacing distances stem from the low cooling rate of the SPS device. 

**Figure 2:**
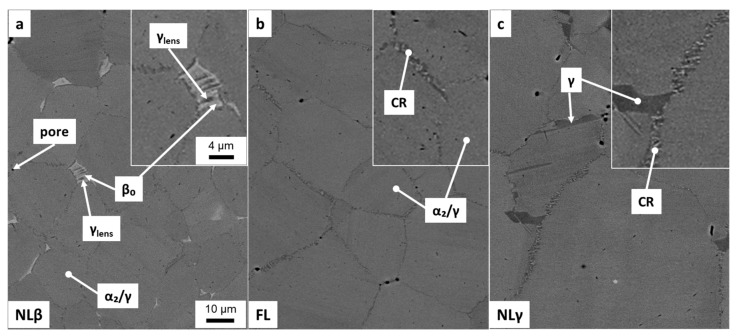
SEM micrographs of microstructures after two-step heat treatment according to Table 1: (**a**) NLβ; (**b**) FL; and (**c**) NLγ. The inserts in the upper right corner show microstructural details in higher magnification. At this point it should be noted that the pores show an enlarged size, caused by the electrolytically etching procedure (see text). SEM images were taken in BSE mode.

Two-step heat treatments were carried out on as-SPS samples to optimize the mechanical properties by adjusting the microstructure, for example, minimizing the lamellar spacing. The first sample experienced a solution heat treatment at 1290 °C for 30 min. This heat treatment takes place within the (α + β)-phase field region, approximately 35 °C above the γ solvus temperature (T_γ,solv_), which is around 1255 °C in case of the TNM alloy [9]. After AC to RT, the samples were aged at 850 °C for 6 h followed by FC to stabilize the microstructure. A detailed record of the heat treatments as well as the quantitative evaluation of the microstructure is summarized in Table 2. Figure 2a shows the resulting microstructure. This microstructure is called NLβ and consists mainly of fine lamellar α_2_/γ-colonies and β_o_-phase at colony boundaries and triple points. When compared to Figure 1a, the lamellar spacing cannot be resolved in this case due to the higher cooling, as achieved by AC. Furthermore, lenticular γ-phase (γ_lens_) precipitates and coarsens within the β_o_-phase during aging at 850 °C for 6 h in order to obtain a thermodynamic phase equilibrium [18]. The amount of γ_lens_ and β_o_-phase was determined to be about 2 vol.%. In addition, so-called cellular reaction (CR) takes place along the colony boundaries. The CR is provoked by the fine lamellae causing a high amount of interface surfaces, thus decreasing the thermal stability of the α_2_/γ-colonies. At aging or service temperature, this fine lamellar α_2_/γ-colonies are prone to decompose in (α_2_ + β_o_ + γ)_cellular_, starting from the colony boundaries that deteriorates the high temperature properties, as reported in References [5,17,26,27].

Furthermore, small pores are also visible inside the SEM micrograph as dark voids, located along the grain boundaries. It should be noted that these pores appear larger in the SEM image than they actually present in the material due to an enlarging effect during the electrolytically etching of the polished surface. Pores, along with the β_o_-phase, which exists as disordered β-phase at 1290 °C, however, impede grain growth during the heat treatment within the (α + β)-phase field region. The mean size of the equiaxed α_2_/γ-colonies was measured by image analysis to be around 20 µm, which corresponds to the as-SPS condition. 

The next heat treatment was carried out at 1265 °C for 30 min, followed by AC and an aging step at 850 °C for 6 h and FC. As a result, the globular γ- and β_o_-phase fraction is decreased to a minimum and an almost FL microstructure could be achieved, which is depicted in Figure 2b. Pores and CR appear along the α_2_/γ-colony boundaries almost in the same manner as they were detected in the previous heat-treated microstructure, see Figure 2a. The colony size of the FL microstructure increases slightly compared to the NLβ one, see Table 2. This is attributed to the low amount of a second phase, which is necessary to effectively inhibit grain coarsening during annealing at 1265 °C. Nevertheless, due to the short holding time of 30 min, the grain size is below 100 µm, which is postulated to be small enough to achieve moderate fracture elongation at RT [1].

The third heat treatment started with an annealing step at 1290 °C for 30 min, as in the first heat treatment, providing a homogeneous (α + β)-microstructure, but afterwards the samples were FC below T_γ,solv_ to 1245 °C and held there for additional 30 min to precipitate globular γ-phase. The following AC and aging corresponds to the previous ones. The resulting microstructure, called NLγ, is shown in Figure 2c. Besides the globular γ-phase, α_2_/γ-colonies, CR and pores are detectable. During the longer first heat treatment step, the former α-grains have grown resulting in a mean grain size of 45 µm. All phase fractions and grain sizes, which were determined via image analysis, are summarized in Table 2. The quantitative image analysis is used to differ between the morphological characteristics of the phases. However, due to the limited resolution of this technique, fine lamellae within the α_2_/γ colonies cannot be evaluated. In contrast, XRD as a lattice-sensitive method leads to the overall phase fraction of α_2_, β_o_ and γ, see Table 2. The heat-treated samples show almost the same phase distribution with around 70 vol.% of γ-phase due to the stabilization annealing at 850 °C. Comparing the XRD measured γ-phase fraction with the globular γ-phase detected by image analysis, it is evident that the majority of the γ-phase is in the form of lamellae within the α_2_/γ-colonies. These α_2_/γ-lamellae are already visible in the as-SPS microstructure (Figure 1a). The lamellar spacing of the colonies can be investigated in more detail by means of TEM, as shown in Figure 1d. The lamellar spacing of the as-SPS sample was determined to be 83 ± 7 nm, see chapter 2. By the subsequent heat treatments, the average lamellar spacing could be reduced by a factor of ten. The average lamellar spacings are almost the same for the NLβ, FL, and NLγ microstructures, see Table 2, due to the fact that the cooling rate as well as the following stabilization heat treatment, which defines the lamellar distance, were the same.

### 3.2. Mechanical Testing

The obtained Vickers hardness of the different microstructures is also summarized in Table 2. The hardness values reflect the microstructural aspects described in the previous chapter. The as-SPS condition shows the lowest hardness with around 378 HV10, due to the coarse lamellae and the highest volume fraction of globular γ-phase, see Table 2. Decreasing the average lamellar spacing by subsequent heat treatments increases the hardness up to 450-470 HV10. For the heat-treated samples the same lamellar width was measured, but the NLγ microstructure shows the lowest hardness value (445 HV10), probably due to the larger colony size and the existence of globular γ-phase. 

**Figure 3:**
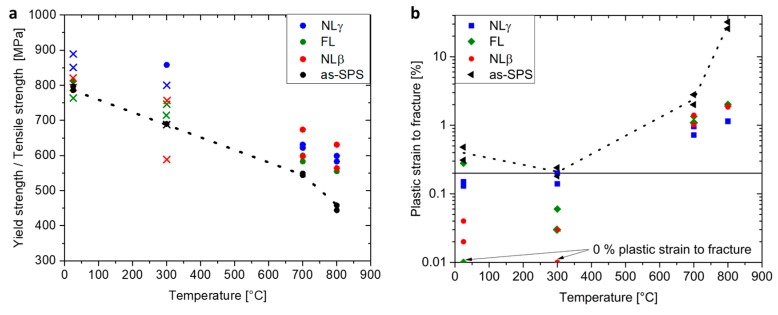
Tensile properties of the investigated material conditions, see Table 2: (**a**) the 0.2%-yield strength is marked with dots, whereas the ultimate tensile strength is denoted with crosses; (**b**) plastic strain to fracture. The 0.2% fracture elongation is indicated as a horizontal line. Two samples failed within the elastic regime. Their values are plotted at the bottom in the logarithmic diagram. The black dashed lines in (**a**) and (**b**) are the baseline provided by the as-SPS condition. For the interpretation of the references to color in this figure legend, the reader is referred to the online version of this article.

For a detailed investigation of the mechanical properties, tensile tests were carried out at 25, 300, 700, and 800 °C. If 0.2% plastic elongation is achieved, the R_p0.2_ yield strength was evaluated (round icons inside Figure 3a). Otherwise, the ultimate tensile strength was determined (marked with a cross). The results are summarized in Figure 3a. The black dashed line represents the baseline provided by the as-SPS data, in order to establish the benefit of the heat treatment. In addition, Figure 3b shows the plastic strain to fracture. It becomes evident that the strength can be increased by heat treatments, due to reduced amount of globular phases and a finer lamellar spacing. However, the plastic strain to fracture decreases by the heat treatment as shown in Figure 3b. This values are always below the black dashed line provided by the as-SPS condition. In addition, in two cases, the plastic strain to fracture decreases to 0%, which is also indicated in the logarithmic diagram in Figure 3b. In summary, the results in Figure 3 show that the strength can be increased by additional heat treatment at 700 and 800 °C, while maintaining a certain ductility, too. 

**Figure 4:**
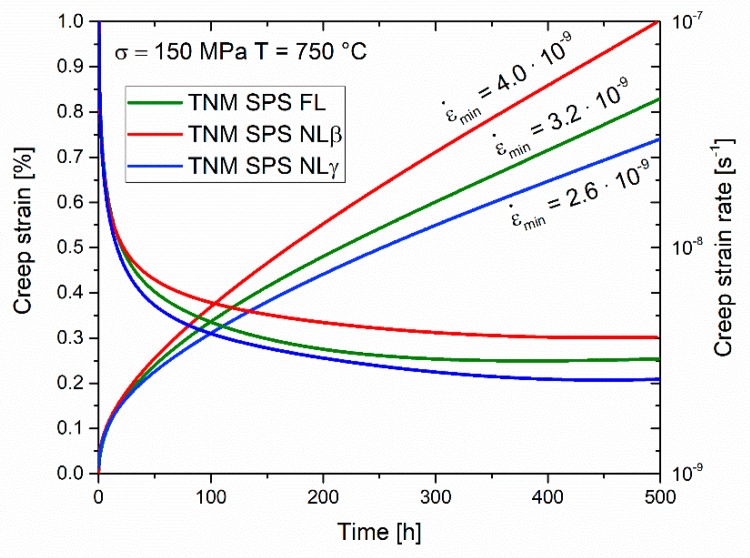
Creep strain and creep strain rate as a function of time of the heat-treated microstructures as a function of time at 750 °C and 150 MPa.

Finally, creep tests were carried out at 750 °C using a load of 150 MPa. The creep tests provided the creep strain (ε) and the creep strain rate ($\dot{\varepsilon }$) as a function of time. The corresponding graphs are plotted in Figure 4. The NLβ microstructure results in a minimum creep rate ($\dot{\varepsilon }{}_{\min}$) of 4.0·10^−9^ s^−1^ after 400 h, the FL state reaches $\dot{\varepsilon }{}_{\min}$ = 3.2·10^−9^ s^−1^ after 350 h and the NLγ variant shows a $\dot{\varepsilon }{}_{\min}$ of 2.6·10^−9^ s^−1^ after 400 h. That means that all tested conditions exhibit a creep strain ≤1% after 500 h at 750 °C.

## 4. Discussion

This study proved that it is possible to manufacture the engineering TNM alloy via SPS by sintering the powder at 1300 °C within the (α + β)-phase field region. A similar fundamental study on the TNM alloy was conducted by Voisin et al. [8]. These authors showed that, at a dwell temperature of 1304 °C, the microstructure consists of α-phase surrounded by β-phase, which is also in agreement with the TNM phase diagram provided by Schwaighofer et al. [9]. During cooling from dwell temperature, a microstructure consisting of coarse lamellar α_2_/γ-colonies, which are surrounded by globular γ- and β_o_-phase was generated in the same way as in this investigation. But in this study, a subsequent heat treatment was designed for the SPS samples to exceed the mechanical results provided by Voisin et al. [8] and reach the full potential of the TNM PM route. The initial as-SPS condition exhibits a NLγ + β_o_ microstructure as shown in Figure 1a, which forms at a dwell temperature of 1300 °C and during slow cooling, whereby globular γ-phase precipitates in the β/β_o_-phase below the T_γ,solv_ temperature. Another transformation, which takes place during cooling, is the formation of lamellar α_2_/γ-colonies. As a consequence of the slow cooling rate, the average lamellar spacing is rather broad, which is associated with a negative influence on the tensile properties [10,13,14]. However, hardness values of 378 HV10 can be reached due to the fine grained microstructure of the as-SPS condition and the elevated content of oxygen, see Table 1, which acts as solid solution hardening element in γ-TiAl alloys [28,29]. As a consequence, the hardness is significantly higher than in a cast/HIP TNM alloy [9,18]. Comparing the tensile tests of this study with as-SPS results from literature [8], similar yield strengths are obtained, but a higher plastic strain to fracture have been reported for the TNM alloy. Obviously, The enhanced oxygen content embrittles the material [28,29], resulting in a deterioration of the fracture elongation. Due to the limited cooling rate of currently used SPS units [8], an optimized microstructure with balanced mechanical properties, i.e., sufficient ductility at RT and good creep resistance at elevated temperatures, as described in Reference [9], cannot be adjusted. Therefore, additional two-step heat treatments were carried out on SPS manufactured samples to remove the coarse lamellae with the adjustment of a NLγ, FL and NLβ microstructure. At this point, it should be noted that in a nominal TNM alloy, no FL microstructure can be adjusted [9]. However, due to the higher amount of oxygen, which stabilizes the α/α_2_-phase [28,30], the appearance of β_o_- and γ-phase could almost be suppressed throughout the heat treatment. The heat treatments are summarized in Table 2 and the resulting microstructures are shown in Figure 2. The hardness values, see Table 2, reflect the arrangement of the constituting phases in these different microstructures. The NLγ sample (Figure 2c) has the lowest hardness, due to its higher volume fraction of the “soft” globular γ-phase and its comparable large α_2_/γ-colony size. In contrast, FL and NLβ exhibits no globular γ-phase, but show almost the same hardness values. Therefore, two opposite effects control the hardness: the high hardness of the β_o_-phase at RT [10,18], whereas the slightly higher colony size of the NLβ microstructure believed to decreases it. Nevertheless, the hardness values of the three heat-treated microstructures show an increase of 70–90 HV10 when compared to the as-SPS microstructure. This behavior is mainly caused by the decreased average lamellar spacing (from 80 nm to 10 nm). The impact of the microstructure on the mechanical properties was also evaluated by tensile tests as summarized in Figure 3. Due to the increased strength of the heat-treated specimens, the ductility is decreased when compared to the as-SPS condition. Therefore, the evaluation of the yield strength at 0.2% plastic elongation was limited for testing temperatures below the ductile-to-brittle transition temperature and thus the ultimate tensile strength values were taken into account. The NLγ microstructures shows the highest ultimate tensile strength (Figure 3a), together with a high fracture elongation (Figure 3b), due to the presence of the rather “soft” globular γ-phase. At 700 °C and 800 °C the strength is enhanced significantly for all heat-treated specimens when compared to the as-SPS condition. The obtained values are in the same range as for a FL microstructure provided by conventional casting and subsequent heat treatment [9]. However, the fracture elongation is lower at RT. Therefore, it is tempting to speculate that the observed embrittlement is due to the increased oxygen content and the appearance of micropores, which are evidenced in Figure 2. In general, these spherical pores appear during the subsequent pressureless heat treatment around the sinter temperature. This effect is called thermally induced porosity (TIP) [31]. The Ar stems from entrapped atomization gas inside the powder, which gets more likely for large powder sizes [19,32] as also used in this study. From Figure 3b it seems that there is a week minimum at 300 °C. Such a minimum might be attributed to strain ageing, i.e., the dislocations are locked by mobile oxygen atoms. Such an embrittlement is also found in many metallic alloy systems. Therefore, in an ongoing project, this effect will be studied by in-situ TEM investigations. 

The different constituting phases in the heat-treated microstructures impact the creep properties as shown in Figure 4. The FL microstructure shows a better creep resistance than the NLβ variant despite same average lamellar spacings. This is caused by the negative effect of the β_o_-phase with B2 structure, which exhibits higher diffusion rates [10,17,33]. In addition, the distinctly smaller colony size of the NLβ microstructure facilitates grain boundary sliding at the chosen creep testing conditions [10,34]. Therefore, as a result of their largest mean colony size, the NLγ microstructure shows the lowest $\dot{\varepsilon }{}_{\min}$, despite of the presence of the soft, globular γ-phase along the colony grain boundaries and same average lamellar spacing. In summary, all heat-treated microstructures shows a $\dot{\varepsilon }{}_{\min}$ within the same magnitude than observed for comparable microstructures produced via the conventional cast and heat treatment route [9], which again highlights the potential of SPS material when an additional heat treatment is performed.

## 5. Summary

In the framework of this study, spark plasma sintering, a technique which is capable of manufacturing complexly shaped components, was chosen to densify powders of an advanced intermetallic γ-TiAl based alloy, the so-called TNM alloy, with a chemical composition of Ti-43.5Al-4.1Nb-1.0Mo-0.1B. Therein, gas atomized powder was sintered at 1300 °C followed by cooling to RT with around 90 K/min. The as-SPS condition reveals a small sized and homogenous NLγ + β_o_ microstructure. At RT the microstructure shows a hardness of 378 HV10 and an ultimate tensile strength of about 800 MPa due to small α_2_/γ-colonies and an elevated oxygen content of 1300 ppm. The average lamellar spacing within the colonies was determined to be 83 ± 7 nm. Subsequent two-step heat treatments shifted the amount of constituting phases during the adjustment of a NLγ, FL and NLβ microstructure, also decreasing the average lamellar spacing to 10 ± 3 nm in order to utilize the full potential of the TNM alloy. As a consequence, the hardness could be increased to 466 ± 4 HV10 in case of the NLβ variant, however, tensile fracture elongation at RT decreased. At 800 °C the NLβ microstructure attain a yield strength of around 600 MPa when compared to the 450 MPa of the initial as-SPS variant. Furthermore, a minimum creep rate of 4.0·10^−9^ s^−1^ was achieved due to the fine lamellar NLβ microstructure. The FL and the NLγ microstructure show lower $\dot{\varepsilon }{}_{\min}$ of 3.2·10^−9^ and 2.6·10^−9^ s^−1^, respectively, which was achieved by the larger mean colony size of 29 and 45 µm, when compared to 20 µm in the NLβ microstructure as well as the absence of a the β_o_-phase.

Summarized, the strength and the creep performance at 750 °C can keep up with the obtained levels of conventional cast and heat treatment processing routes [9]. Therefore, the study proves that SPS of the TNM alloy, along with a designed subsequent heat treatment, is a promising alternative manufacturing route besides the conventional processing of engineering γ-TiAl based alloys by means of investment casting.

## Figures and Tables

**Table 1:** Chemical composition of the investigated TNM alloy in at.% after SPS. The oxygen content is stated in mass-ppm.

Ti	Al	Nb	Mo	B	O
bal.	43.45	4.05	1.02	0.10	1300

**Table 2:** Microstructural state in as-SPS and heat-treated condition (see text).

Type of Microstructure	Heat Treatment	Phase Fractions Measured via XRD *			Quantitative Morphological Analysis of SEM Micrographs **				Lamellar Spacing	Hardness
		α_2_	γ	β_o_	α_2_/γ-colonies		globular γ	β_o_+γ_lens_		
		[vol.%]	[vol.%]	[vol.%]	fraction [vol.%]	size [µm]	fraction [vol.%]	fraction [vol.%]	[nm]	[HV10]
as-SPS	-	35	61	4	94	19	5	<1	83 ± 7	378 ± 8
NLβ	1290 °C/30 min/AC + 850 °C/6 h/FC	27	71	≤2	97	20	<1	2	8 ± 9	466 ± 4
FL	1265 °C/ 30 min/AC + 850 °C/6 h/FC	30	68	≤2	98	29	<1	<1	10 ± 6	461 ± 7
NLγ	1290 °C/30 min/FC 1245 °C/30 min/AC + 850 °C/6 h/FC	30	68	≤2	97	45	2	<1	9 ± 8	445 ± 5

* The estimated accuracy of the method is ± 2 vol.%. ** The estimated accuracy is ± 1 vol.%.
